# Effects of plyometric-based high-intensity interval training on muscle-to-fat ratio in children: protocol for a randomized controlled trial in primary school settings

**DOI:** 10.1007/s12519-026-01064-z

**Published:** 2026-07-18

**Authors:** Ying Liu, Bjorn T. Tam, Sin-Yee Chan, Yu-Ying Wang, Ke-Wen Wan, Wing-Lam Chung, Asefa Adimasu Taddese, Ka-Yin Chan

**Affiliations:** 1 Academy of Wellness and Human Development, Faculty of Arts and Social Sciences, Hong Kong Baptist University, Kowloon Tong, Hong Kong SAR China; 2 Dr. Stephen Hui Research Centre for Physical Recreation and Wellness, Hong Kong Baptist University, Kowloon Tong, Hong Kong SAR China

**Keywords:** Childhood obesity, High-intensity interval training, Muscle-to-fat ratio, Plyometric training, School-based intervention

## Abstract

**Background:**

Childhood obesity is associated with cardiometabolic and musculoskeletal health risks. Because fat reduction may be accompanied by loss of skeletal muscle, this trial aims to evaluate whether a plyometric-based high-intensity interval training (Plyo-HIIT) program improves muscle-to-fat ratio (MFR) in children aged 9–12 years.

**Methods:**

This pragmatic, assessor-blinded, three-arm randomized controlled trial will recruit 210 children aged 9–12 years from primary schools in Hong Kong, China. Participants will be randomly assigned to Plyo-HIIT, structured fitness (SF) program, or the health education (HE) control group in a 1:1:1 ratio. Each group will engage in two 45-minute weekly sessions for 16 weeks. The Plyo-HIIT program involved developmentally appropriate plyometric exercise (e.g., jumping, skipping). The SF program includes moderate- to high-intensity activities maintaining 60%–80% of maximal heart rate. The HE group will receive weekly health education sessions. Outcomes will be assessed at baseline, post-intervention (16 weeks), and at 6-month follow-up. The primary outcome will be MFR measured using magnetic resonance imaging. Secondary outcomes will include body composition, muscular fitness, cardiorespiratory fitness, flexibility, body mass index, physical activity, and cortisol levels.

**Results:**

Participant recruitment was conducted between September 26, 2024, and March 14, 2025. The 16-week intervention has been completed. However, as one participating cohort delayed the start date of the intervention, the 6-month follow-up assessments are still ongoing, with the final follow-up assessment scheduled for August 2026. No trial outcome data are reported in this protocol article.

**Conclusions:**

This study is expected to provide evidence for the effectiveness of Plyo-HIIT in improving body composition by increasing the muscle-to-fat ratio among school-age children. This will support the integration of Plyo-HIIT training into school-based physical activity programs, offering a practical and time-efficient strategy to prevent early-onset obesity. By comparing Plyo-HIIT with the SF and HE groups, this study may further demonstrate the value of plyometric training for primary school settings.

**Graphical abstract:**

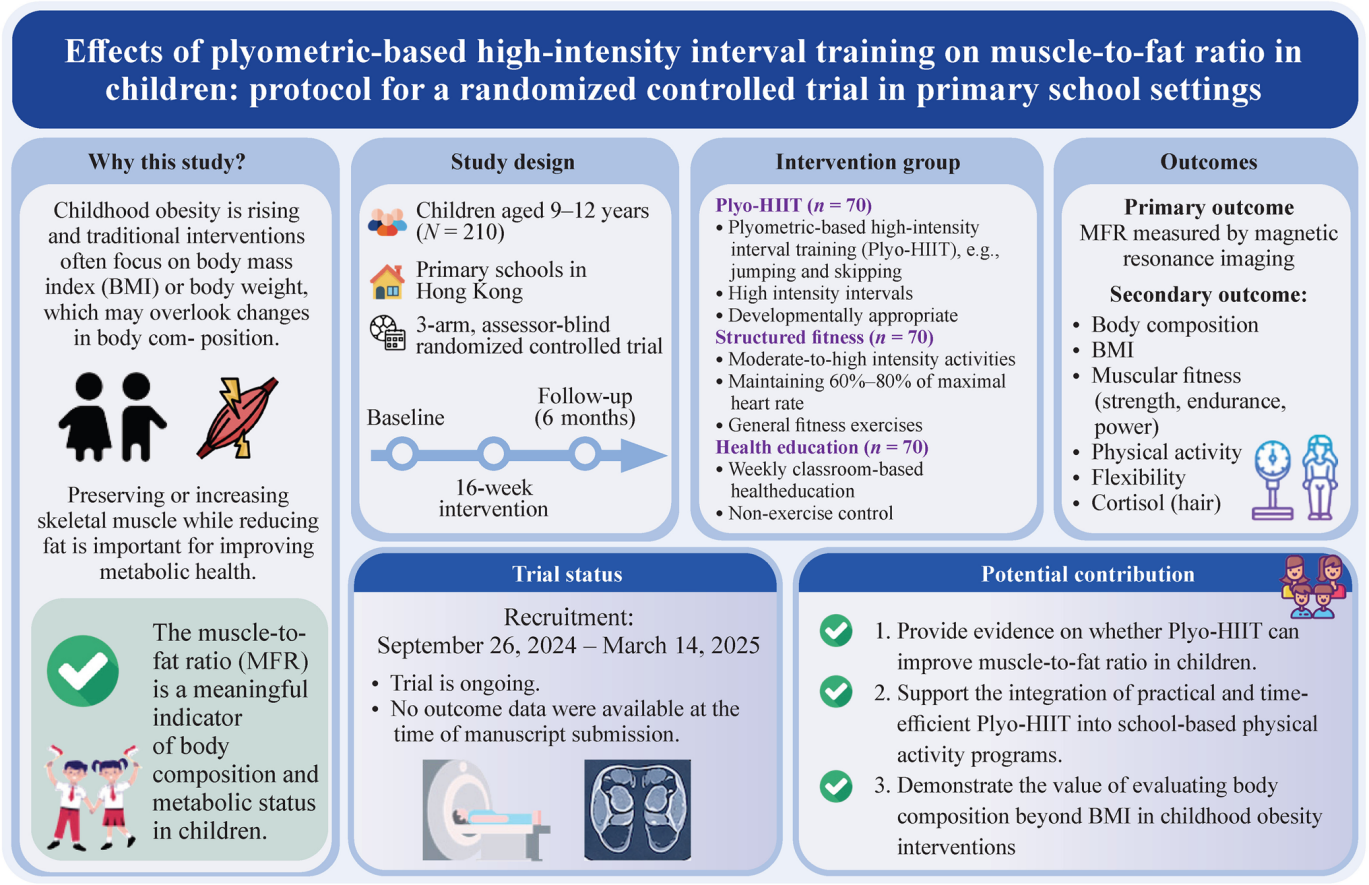

## Introduction

Childhood obesity is a global public health concern characterized by excessive accumulation of body fat that negatively affects the health and well-being of children. The World Health Organization (WHO) reports that the global prevalence of childhood obesity has increased dramatically, with over 390 million children and adolescents (aged 5–19 years) and an estimated 37 million children under the age of 5 classified as overweight or obese. Childhood obesity is strongly associated with an increased risk of developing chronic disease in adulthood, such as cardiometabolic disorders, musculoskeletal complications, and cancers [1]. In addition to physical health complications, childhood obesity is also associated with significant social and psychological challenges, including poor self-esteem, feelings of loneliness, an elevated risk of depression, and social stigmatization [2, 3]. These factors can seriously impact a child’s quality of life and psychosocial development. However, many of these associated physical and psychological challenges can be effectively mitigated by adopting and maintaining an active and health-conscious lifestyle.

Traditional physical activity interventions targeting pediatric obesity typically emphasize aerobic exercise, strength training, and recreational sports, with the primary aim of improving energy balance and reducing excess adiposity. However, these approaches often pay more attention to managing body mass index (BMI) through caloric restriction and increased physical activity, which may result in the loss of both fat and muscle mass. This loss of muscle, in particular, can negatively impact metabolic health and physical function [4, 5]. Skeletal muscle plays a critical role in metabolic homeostasis, serving as a primary site for glucose uptake and amino acid storage [6, 7]. Loss of muscle mass can reduce the availability of protein and energy substrates, which negatively affects recovery from illness, slows wound healing, and reduces basal metabolic rate [8]. In addition, skeletal muscle contributes approximately 20%–30% of total resting energy expenditure (REE), indicating its important role in maintaining energy balance [9]. Reduction in muscle mass may therefore lower REE, impair metabolic function, and increase the likelihood of fat accumulation and weight regain over time [6, 10, 11]. These limitations suggest that evaluating intervention effectiveness through BMI alone may overlook meaningful improvements in body composition and metabolic health.

Muscle-to-fat ratio (MFR), defined as the proportion of skeletal muscle mass relative to fat mass, has recently been considered as a critical indicator of metabolic health [12–14]. Unlike BMI, which reflects total body mass relative to height but does not distinguish between lean and adipose tissue, MFR is capable of balancing the relationship between metabolically protective and metabolically detrimental compartments. Skeletal muscle serves as a primary site for glucose disposal and insulin-mediated metabolic regulation, whereas excess adipose tissue is associated with systemic inflammation and cardiometabolic dysfunction [7, 15, 16]. Therefore, the relative proportions of these tissues may provide a more physiologically informative representation of metabolic status than total body weight alone. Emerging evidence suggests that lower MFR is associated with increased cardiometabolic risk, reduced physical function, and higher likelihood of metabolic syndrome in both pediatric and adult populations [12, 17]. Importantly, during growth and maturation in childhood, improvements in body composition may not always be reflected by reductions in BMI. Exercise interventions that reduce fat mass while preserving or enhancing muscle mass may bring significant metabolic benefits even in the absence of substantial BMI change. Given this relationship, interventions aimed at reducing fat mass must also prioritize the preservation of muscle mass, particularly in the pediatric population, to maintain metabolic health and prevent long-term complications.

Exercise modalities that simultaneously promote neuromuscular development and metabolic adaptation may be particularly advantageous in children. Plyometric exercise involves repeated stretch-shortening cycle actions, such as jumping, hopping, and bounding, which can stimulate the development of neuromuscular coordination, strength, and power [18, 19]. Evidence has indicated that resistance-based and plyometric training can be safe and beneficial for young people when it is implemented in an age-appropriate, progressively structured, and closely supervised way, and is focused on movement quality rather than maximal performance [20]. Beyond neuromuscular benefits, activities based on weight-bearing jumping also generate mechanical loading, which may promote skeletal development during growth. School-based jumping or plyometric programs have been shown to improve bone-related outcomes at key anatomical sites, such as the femoral neck and lumbar spine [21, 22]. These musculoskeletal adaptations further support the inclusion of plyometric components in school-based interventions, provided that training intensity, volume, and progression can be appropriately controlled.

Given these benefits, further investigation into training modalities that combine the musculoskeletal advantages of plyometrics with additional cardiovascular improvements is needed. High-intensity interval training (HIIT) has been recognized as an efficient exercise modality that achieves health benefits through a short, time-efficient session. It involves alternating short bursts of intense exercise with periods of rest or low-intensity activity. The 2020 WHO guidelines for children and adolescents aged 5–17 years recommended that, in addition to achieving at least 60 min of moderate-to-vigorous physical activity daily, vigorous-intensity aerobic activities and those that strengthen muscle and bone should be incorporated at least 3 days per week [23]. These recommendations apply to all children and adolescents regardless of weight status.

Integrating developmentally appropriate plyometric exercise into a plyometric-based high-intensity interval training (Plyo-HIIT) may therefore provide a practical training strategy that is consistent with current international physical activity guidelines. Such a program is not designed as maximal or performance-oriented athletic training. Instead, it emphasizes movement quality, controlled landing mechanics, neuromuscular coordination, and the gradual introduction of jump-based tasks within a supervised school environment. By combining muscle- and bone-strengthening activities with cardiovascular training, Plyo-HIIT may contribute to improvements in lean mass, reductions in adiposity, and overall physical fitness.

To date, however, no randomized controlled trial (RCT) has specifically examined the impact of a school-based Plyo-HIIT program on magnetic resonance imaging (MRI)-derived MFR in children. If effective, the findings may provide evidence to inform future school-based physical activity strategies and policy development for children. Consequently, the study aims to investigate this gap by exploring whether a school-based Plyo-HIIT program can effectively improve body composition among school-age children, particularly when compared to a structured fitness (SF) program and a non-exercise healthy education (HE) program.

## Methods

### Study hypothesis and purpose

This study aimed to investigate the effectiveness of a 16-week school-based Plyo-HIIT intervention in improving MFR among children aged 9–12 years. Specifically, the study compares the outcomes of a Plyo-HIIT program with the SF program and the HE control group. The primary hypothesis is that children allocated to the Plyo-HIIT group will demonstrate greater improvements in MRI-derived MFR compared with the SF and HE groups immediately following the intervention (week 16). The 6-month follow-up assessment (week 42) is included to examine whether intervention effects are maintained over time. In addition, the study will examine intervention-related changes in InBody-derived body fat percentage, muscular strength, endurance and power, cardiorespiratory fitness, flexibility, physical activity levels, body mass index (BMI), and physiological stress, indicated by hair cortisol levels.

### Study design and study setting

This study was designed as a three-arm, parallel-group, assessor-blinded pragmatic RCT to evaluate the effectiveness of a school-based intervention over a 16-week period, with an additional 6-month follow-up. Outcome assessments will be conducted at three time points: baseline (pre-intervention), immediately after the 16-week intervention, and at the 6-month follow-up (week 42). Participants will be randomly allocated to one of three groups: (1) Plyo-HIIT, (2) SF program, or (3) HE control group.

All interventions will be delivered in local primary schools in Hong Kong, China by trained instructors following standardized protocols. Outcome assessments, including MRI acquisition and image analysis, will be conducted by independent assessors who are blinded to group allocation. The overall study flow is listed in Table 1. This study has been reviewed and approved by an institutional research ethics committee (Ref. No.: REC/22-23/0536). Written informed consent will be obtained from the parents or legal guardians of all participants prior to data collection.

**Table 1:** Schedule of enrollment, assessments, and interventions

Items	Enrollment	Baseline (week 0)	Intervention (week 1–16)	Post-intervention (week 16)	Follow-up (week 42)
Enrollment					
Eligibility screen	✓				
Informed consent (participant + parent)	✓				
Allocation to 3 groups		✓			
Interventions^a^					
Health education control			✓		
Structured fitness program			✓		
Plyo-HIIT program			✓		
Assessments					
MRI		✓		✓	✓
InBody		✓		✓	✓
Seated leg press and handgrip strength		✓		✓	✓
Static curl-up and plank		✓		✓	✓
Vertical jump and squat jump		✓		✓	✓
20 m shuttle run		✓		✓	✓
Sit and reach		✓		✓	✓
BMI		✓		✓	✓
PAQ-C questionnaire		✓		✓	✓
Hair cortisol sampling		✓		✓	✓

*BMI* body mass index, *InBody* bioelectrical impedance analysis-based body composition assessment, *MRI* magnetic resonance imaging, *PAQ-C* Physical Activity Questionnaire for Older Children, *Plyo-HIIT* plyometric-based high-intensity interval training

^a^The three intervention arms were delivered twice weekly during weeks 1–16. The symbol “✓” indicates that the procedure, intervention, or assessment is conducted at the corresponding study time point

### Participants and eligibility

A total of 210 healthy children, aged 9–12 years, will be recruited from local primary schools in Hong Kong, China. Participants must meet the following inclusion criteria: (1) age between 9 and 12 years; (2) ability to understand and communicate effectively in Chinese; and (3) attending local primary schools in Hong Kong, China.

Exclusion criteria are: (1) inherent or serious diseases that limit the ability to participate in physical activity, such as congenital heart disease, pediatric cancer, or Down syndrome; (2) diagnosed mental disorders that can impair daily behavior and functioning, such as depression, anxiety disorder, attention-deficit/hyperactivity disorder, autistic spectrum disorder, or psychotic disorder; (3) physical disabilities that require assistive devices for walking; (4) impaired vision or hearing; (5) intellectual disabilities or cognitive deficits requiring special care and educational needs; (6) currently participating in a structured exercise program or organized sports training more than twice weekly; and (7) musculoskeletal injuries experienced within the past 6 months. A summary of the inclusion and exclusion criteria is provided in Table 2.

**Table 2:** Study inclusion and exclusion criteria

Inclusion criteria	Exclusion criteria
1. Aged between 9 and 12 y	1. Inherent or serious diseases limiting physical activity (e.g., congenital heart disease, pediatric cancer, Down syndrome)
2. Able to understand and communicate effectively in Chinese	2. Diagnosed with mental illness that impair behavior and functioning (e.g., depression, anxiety disorder, attention-deficit/hyperactivity disorder, autistic spectrum disorder, psychotic disorder)
3. Attending local primary schools in Hong Kong, China	3. Physical disabilities requiring assistive devices for walking
	4. Impaired vision or hearing
	5. Intellectual disabilities or cognitive deficits requiring special care or educational needs
	6. Currently participating in a structured exercise program or organized sports training more than twice weekly 7. Experienced musculoskeletal injuries within the past 6 mon

### Randomization and blinding

**Fig. 1:**
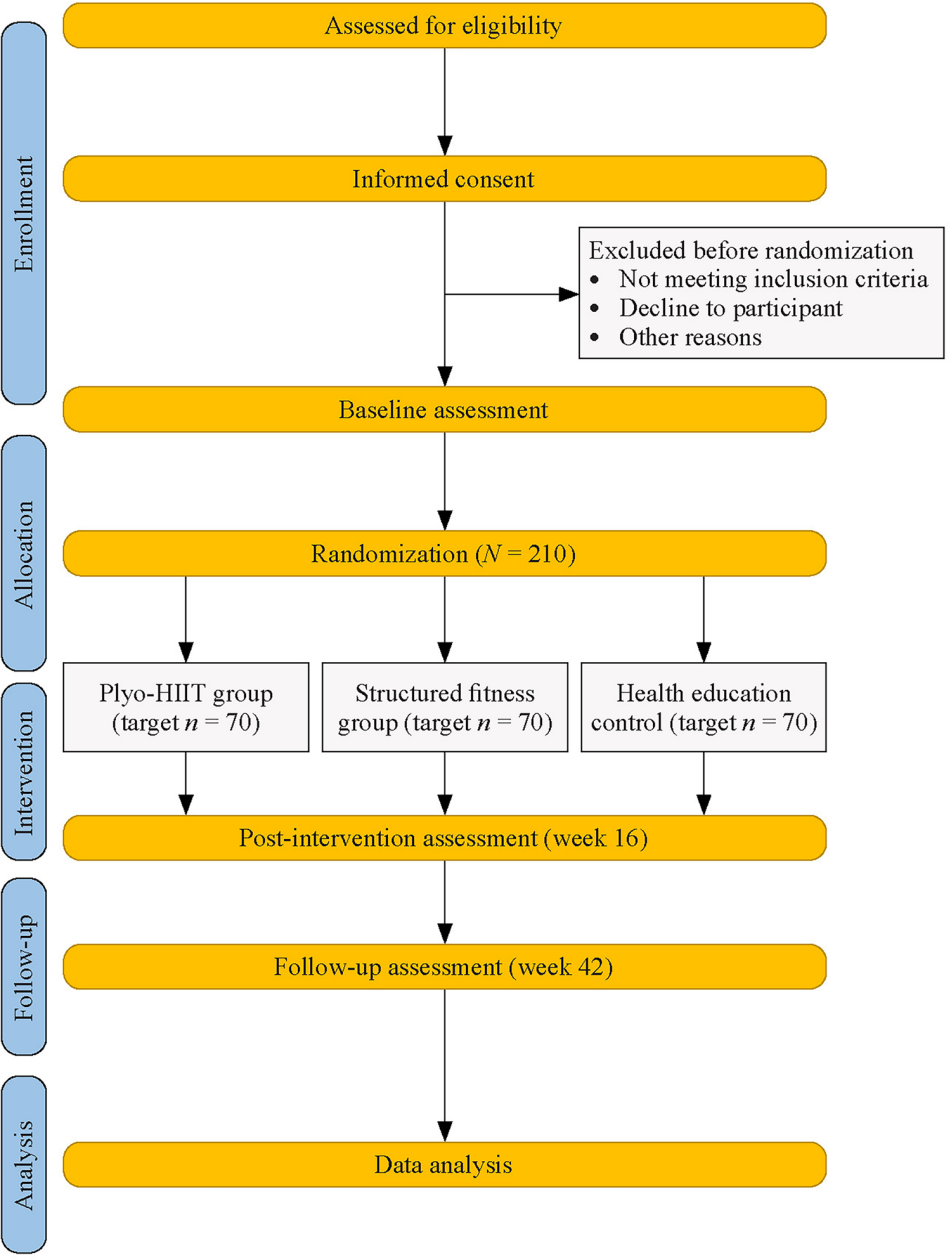
Flow diagram for participant screening, randomization, assessments, and interventions. This figure presents the planned participant flow and does not represent finalized participant flow data. The final numbers of participants screened, excluded, randomized, and retained at each assessment will be reported after trial completion. The planned target sample size is 210 participants, with approximately 70 participants allocated to each group. *Plyo-HIIT*, plyometric-based high-intensity interval training

Participants will be randomly assigned to one of the three study groups (Plyo-HIIT, SF program, or HE control) using a computer-generated randomization list with randomly permuted block sizes of 3 and 6 to achieve a 1:1:1 allocation ratio (Fig. 1). The allocation sequence will be generated by an independent researcher who is not involved in recruitment, assessment, or intervention delivery to prevent allocation bias. Group allocation will be concealed using sealed, opaque, and consecutively numbered envelopes prepared by an independent researcher to ensure allocation concealment until the point of assignment.

Due to the nature of the intervention, participants, exercise instructors, and research personnel cannot be blinded. Therefore, the study is designed as an assessor-blinded randomized controlled trial. MRI acquisition, image segmentation, body composition assessments, and statistical analyses will also be performed by personnel blinded to group allocation. Participants will be instructed not to reveal their group allocation during outcome assessments.

### Interventions

This study consists of three intervention groups: Plyo-HIIT, SF program, and HE control group. All interventions will be delivered over a 16-week period, with two 45-minute sessions per week during school-arranged after-school activity periods at participating schools. To support adherence and minimize loss to follow-up within a school-based setting, sessions will be scheduled as part of regular school activities and delivered in groups, with attendance recorded throughout the intervention period. The SF and Plyo-HIIT interventions will be delivered by two to three trained instructors who have completed a standardized training session. Intervention fidelity will be maintained through standardized instructor training, class video recordings, and biweekly on-site monitoring by researchers to verify protocol delivery and provide feedback when needed.

The Plyo-HIIT group will receive a progressive exercise program that integrates developmentally adapted plyometric drills within a structured HIIT framework. The intervention is not designed as maximal or performance-oriented athletic plyometric conditioning. Instead, it aims to improve neuromuscular strength, lean mass development, and cardiometabolic efficiency while emphasizing movement quality, controlled landing mechanics, and coordinated performance. Each session will consist of a standardized 10-minute warm-up, the main exercise phase, and an active cooldown. The main exercise phase will begin with four 2-minute rope skipping intervals, each followed by a 1-minute active recovery period. Participants will then perform structured blocks of plyometric-based circuits, with exercise complexity and volume progressing across the program. Weeks 1–4 will serve as an adaptation phase to facilitate safe familiarization with movement patterns and loading. During this phase, participants will perform one to two blocks including double-leg jumps, medicine ball dips, and burpees. In weeks 5–8, participants will perform two blocks of hurdle hops, zigzag jump drills, and plank jacks. In weeks 9–12, two blocks of split squat jumps, tuck jumps, and mountain climbing will be performed. In weeks 13–16, two blocks of single-leg cone hops, single-leg zigzag drills, and caterpillar walks combined with plank jacks will be performed [24]. Within each block, exercises will be performed using a fixed interval format of 15 seconds of activity followed by 15 seconds of passive recovery, with a 1-minute passive rest between blocks. This work–rest pattern, combined with standardized training sequences and gradual increases in jumping contacts, will be used to regulate training load and reduce fatigue-related technique deterioration in a pragmatic school setting where continuous physiological monitoring may not be feasible for all sessions. Jump contact volume will be progressively increased from approximately 50 contacts in week 1 to approximately 80 contacts by week 13. This progression will be implemented conservatively to control mechanical loading, maintain movement quality, and reduce the risk of overuse or fatigue-related injuries. All Plyo-HIIT sessions will be delivered under close supervision by trained instructors, who may adjust repetitions and rest intervals to maintain safe technique and accommodate individual tolerance while preserving the overall training structure. Children who attend at least 80% of sessions will receive an attendance certificate to promote engagement.

Participants assigned to the SF group will follow a general structured fitness program aligned with the American College of Sports Medicine and WHO recommendations for youth physical activity. Each session will begin with a 10-minute warm-up that includes jogging and static stretching targeting major muscle groups. This will be followed by approximately 30 minutes of moderate- to high-intensity physical activity, including sport-based games such as basketball, football, and sprinting drills, as well as obstacle courses, dance routines, and basic motor skill drills. The target exercise intensity will be maintained at 60%–80% of age-adjusted maximal heart rate, monitored using Polar heart rate sensors worn by each participant. Sessions will conclude with a 5-minute cooldown involving static stretching and light movement. The first 4 weeks will serve as an adaptation period to gradually introduce participants to structured exercise.

Participants assigned to the HE control group will receive classroom-based health education. Each session will last approximately 45 min and will be delivered by trained researchers in a small-group setting. The course will cover core themes related to physical activity, dietary behaviors, and mental health, incorporating interactive discussions and classroom games to enhance engagement. This group will serve as a non-exercise comparator and will enable the isolation of treatment effects attributable to physical activity.

### Outcome measures

#### Primary outcome: muscle-to-fat ratio

MFR will be evaluated using MRI, serving as the primary outcome of intervention effects. The primary endpoint is the between-group difference in change in MRI-derived MFR from baseline to immediately post-intervention (week 16). The 6-month follow-up assessment will be used to examine the maintenance of intervention effects over time. MRI assessments will be conducted at baseline, post-intervention, and 6-month follow-up.

The MRI assessments will be conducted using a 3 Tesla MRI system (MAGNETOM Prisma, Siemens Healthineers, Erlangen, Germany) with an 18-channel body coil and a 32-channel spine coil. A three-point Dixon water–fat separation technique will be implemented using a T1-weighted volumetric interpolated breath-hold examination (T1 VIBE Dixon) sequence to separately quantify muscle and fat tissue volumes [25].

Two anatomical regions will be examined: (1) the abdominal region, with axial slices centered at the L4–L5 intervertebral disc [26], and (2) the mid-thigh, located at the midpoint between the anterior superior iliac spine and the upper end of the patella [27].

A focused body composition acquisition protocol will be used rather than a full diagnostic scan to reduce acquisition time and participant burden. The total MRI acquisition time for the body composition protocol is approximately 30 min per participant, including positioning and sequence acquisition. The same imaging parameters will be applied across time points, with anatomical positioning standardized according to predefined landmarks. Acquisition parameters will include a repetition time (TR) of 4.0 ms, first echo time (TE1) of 1.29 ms, flip angle of 9°, slice thickness of 3.5 mm, and a field of view (FOV) read as 380 × 344 mm. Images will be reconstructed into water-only, fat-only, in-phase, and out-of-phase datasets for subsequent tissue segmentation.

Image segmentation and quantification will be performed using 3D slicer software (Version 4.11). Skeletal muscle and adipose tissue compartments will be segmented semi-automatically with manual correction where necessary. MFR will be calculated as follows: MFR = skeletal muscle volume (cm^3^)/total adipose tissue volume (cm^3^).

All MRI acquisition and image analyses will be conducted by trained personnel blinded to group allocation. To enhance measurement reliability, a random subset (10%) of scans will be re-analyzed to assess intra-rater consistency.

#### Secondary outcomes

##### Percentage body fat

The percentage body fat will be determined using the InBody body composition analyzer (InBody 770, InBody Co., Seoul, Korea). Participants will be required to stand barefoot on the device and grip the hand electrodes in a standardized posture for measurement. To minimize measurement errors, assessments will be performed with participants wearing light clothing and at least 2 hours postprandially. The InBody 770 provides detailed body composition analysis, including fat mass, lean body mass, and percentage body fat. Results will be automatically calculated and recorded. Duplicate measurements will be taken. If values differ by > 1.0% body fat, a third measurement will be performed and the median recorded [28].

##### Change in muscular strength

The muscular strength will be assessed using handgrip, measured with a digital dynamometer (Takei Physical Fitness Test GRIP-A, T.K.K.5001, Takei Scientific Instruments Co., Ltd., Japan). Participants will stand with their arm by their side without elbow flexion and squeeze the device with maximum effort for 2–3 seconds. Three trials per hand with 10-second of rest between trials will be performed. The highest value either from the right or left hand will be recorded for analysis; the mean of the best right/left trials will be retained for quality control [29, 30].

##### Change in muscular endurance

Static curl-up and prone plank tests will be used to assess muscular endurance [31–33]. In the static curl-up test, participants will assume a curl-up position with the torso flexed at 45°. They will maintain this posture for as long as possible, and the total time (in seconds) sustained in the correct position will be recorded. If the posture is lost for more than 3 seconds, the test will be considered as terminated.

In the prone plank test, participants will support their body on forearms and toes on a mat while maintaining a straight line from head to heels. No floor contact will be allowed other than forearms and toes. Time to failure will be recorded.

##### Change in muscular power

Muscular power will be assessed using two jump-based tests: the vertical jump and the squat jump [34–37]. Both tests will be measured using equipment from Swift Performance Equipment (Queensland, Australia).

For the vertical jump, a Vertec-style jump measurement device will be used. This test consists of a stack of horizontal vanes that can be displaced when touched, allowing direct measurement of maximal reach height. Participants will stand with feet shoulder-width apart and perform a maximal upward jump using both arms and legs to optimize height. Jump height will be calculated as the difference between standing reach height and maximum jump reach. Two trials will be conducted, and the best height (in cm) will be used for analysis.

In the squat jump, a contact mat system will be used to record flight time and calculate jump height using standard kinematic equations. Participants will begin from a static squat position (knee angle 90°, hands on hips, torso upright) and jump vertically without countermovement or arm swing. Two trials will be performed, and the best result will be recorded.

##### Change in cardiorespiratory fitness

Cardiorespiratory fitness will be assessed using the 20-m shuttle run test [38–40]. Participants will run continuously between two markers 20 m apart, guided by audio beeps. The frequency of beeps increases incrementally. The test is terminated if the participant fails to reach the marker twice consecutively or the participant misses the marker three times in total. When the participant can no longer follow the pace, the highest level reached will be used to estimate the maximal oxygen uptake (VO_2_ max).

##### Change in flexibility

Flexibility will be assessed using the sit-and-reach test [41–43]. Participants will sit with their legs fully extended and reach forward along a measuring line while keeping their knees straight. The farthest distance reached will be recorded in centimeters.

##### Change in body mass index

BMI will be calculated by dividing the body mass (kg) by height squared (m^2^). Body mass and height will be measured using a calibrated digital scale and stadiometer (to 0.1 kg and 0.1 cm), with participants barefoot and in light clothing.

##### Change in physical activity

Moderate-to-vigorous physical activity levels will be assessed using the validated Chinese version of the Physical Activity Questionnaire for Older Children (PAQ-C) [44–46]. The questionnaire captures the previous 7 days’ activities; each item is scored 1–5 and averaged to produce a total score. Standard PAQ-C missing-item rules will be applied; questionnaires with > 20% missing will be excluded. This self-report will be used to collect data on physical activity levels.

##### Change in cortisol level

Physical activity interventions have been shown to influence stress regulation and hypothalamic–pituitary–adrenal (HPA) axis function in children and adolescents [47]. Therefore, examining cortisol levels may help explore whether participation in structured exercise programs influences physiological stress regulation in children.

Hair cortisol levels will be used as an indicator of chronic physiological stress and as a psychological marker [48, 49]. A 3-cm segment will be cut from the posterior vertex (20–50 mg) to reflect the prior 3 months of cortisol exposure. Samples will be stored in aluminum foil at room temperature in the dark [50]. Hair washing and cortisol extraction will be performed following a previously established protocol, including isopropanol washing and methanol extraction. Cortisol concentrations will be analyzed using liquid chromatography–tandem mass spectrometry (LC–MS/MS). For internal quality control, 5% of samples will be randomly selected for duplicate analysis to verify reliability [51, 52].

### Sample size

Sample size was calculated based on the primary outcome, MRI-derived MFR, using a repeated-measures analysis including baseline, post-intervention, and 6-month follow-up measurements. The primary endpoint is the between-group difference at the post-intervention assessment (week 16), with the 6-month follow-up included to evaluate maintenance of intervention effects over time.

A power analysis was conducted using G*Power 3.1 (*F* test, repeated-measures ANOVA, within-between interaction; 3 groups × 3 measurements). As no prior randomized controlled trials have examined MRI-derived MFR changes in children following Plyo-HIIT, an effect size specific to MFR is currently unavailable. Therefore, the expected effect size will be estimated based on pediatric exercise intervention studies reporting changes in BMI-related adiposity outcomes, which are conceptually related to body composition adaptations.

A previous study reported a mean between-group difference of 0.2 in BMI *Z* score, with a standard deviation (SD) of 0.45, corresponding to Cohen’s *d* ≈ 0.44 [53]. This was converted to an ANOVA effect size of *f* = 0.22 and used as a conservative proxy for the primary outcome. This approach was adopted to avoid underestimating the required sample size in the absence of directly comparable MFR intervention data.

To adopt a stringent and conservative design strategy, the significance level was set at *α* = 0.01. Statistical power was set at 90% to ensure adequate sensitivity for detecting clinically meaningful intervention effects over time.

Given the 16-week and 6-month follow-ups, a conservative assumption was made for the correlation among repeated measures (*r* = 0.2) to avoid overestimating within-subject stability across extended time intervals. The Greenhouse–Geisser nonsphericity correction was set to *ε* = 0.70, reflecting possible variability and differences between time intervals.

With these assumptions, the analysis indicated that a total of 153 participants is required. To accommodate an estimated 25% attrition rate, the minimum sample size was set at 204. To further ensure adequate power for subgroup and sensitivity analyses, and to allow for potential uneven group sizes after attrition, the recruitment target was rounded up to 210 participants (70 per group).

### Statistical analysis

All statistical analyses will be performed using SPSS version 28.0 (IBM Corporation, Armonk, NY, USA). Descriptive statistics will be used to summarize baseline participant characteristics in each group. Continuous variables (e.g., age, BMI, percentage body fat) will be presented as means and standard deviations, while categorical variables (e.g., sex, group allocation) will be presented as frequencies and percentages.

The primary outcome measure is the MRI-derived MFR. The primary analysis will focus on the between-group difference in change in MFR at the post-intervention assessment (week 16); this is the primary outcome indicator for the trial. The 6-month follow-up assessment will be included to examine the maintenance of intervention effects over time. Generalized estimating equations (GEE) will be used to account for within-subject correlation across the two post-baseline assessments (post-intervention at 16 weeks and 6-month follow-up). The intervention group will be treated as a three-level categorical variable, with the HE control group as the reference. Time will be specified as a categorical variable. Group, time, and group-by-time interaction will be included in the model. Baseline MFR values, age, and sex will be included as covariates. An exchangeable working correlation structure will be specified, with robust sandwich standard errors used for estimation. Pairwise comparisons will be performed to compare Plyo-HIIT with SF and HE at week 16. Sensitivity analyses using a different working correlation structure will also be performed.

Secondary outcomes, including percentage body fat, muscular strength, muscular endurance, muscular power, cardiorespiratory fitness, flexibility, BMI, physical activity level, and cortisol levels, will be analyzed similarly using a GEE model. For each secondary outcome, the corresponding baseline value will be included as a covariate where appropriate. These analyses will be considered exploratory.

All analyses will follow the intention-to-treat principle, including all randomized participants regardless of adherence. Missing data will be processed using multiple imputation methods to minimize bias as much as possible. A per-protocol sensitivity analysis will also be performed including only participants with at least 80% attendance in intervention sessions. Effect estimates will be reported with 95% confidence intervals where appropriate. For the prespecified primary pairwise comparisons, the significance level will follow the adjustment described in the sample size calculation. For exploratory secondary analyses, a two-sided *P* value < 0.05 will be considered statistically significant.

### Data management

To ensure data accuracy and confidentiality, double data entry will be conducted by trained researchers. Each participant will be assigned a unique research ID code to anonymize all records. Personal identifiers will not be entered into the main study database to ensure strict confidentiality.

The research team will continuously monitor data quality at baseline, post-intervention (week 16), and 6-month follow-up. MRI scans, physical assessments, and questionnaire responses will be regularly checked for completeness and consistency. Any protocol deviations, adverse events, or data anomalies will be documented and discussed during research meetings. Any unexpected or repeated adverse events will be reviewed in consultation with a qualified medical professional to determine whether study participation should be discontinued.

All study data will be stored separately from participants’ personal information. Hard copies will be kept in locked filing cabinets, while electronic files will be saved on encrypted, access-controlled hard drives. Only authorized research personnel will have access to these records. Backups will be performed regularly to prevent data loss, and all identifiable information will be stored securely and separately from outcome data.

## Results

This protocol describes an ongoing randomized controlled trial registered in the Chinese Clinical Trial Registry (ChiCTR2500110770). Participant recruitment took place between September 26, 2024 and March 14, 2025. The 16-week intervention has been completed. However, as one participating cohort delayed the start date of the intervention, the 6-month follow-up assessments are still ongoing, with the final follow-up assessment scheduled for August 2026. Data cleaning is being conducted for completed assessments, and final outcome analysis will be performed after completion of the remaining follow-up assessment. Therefore, no trial outcome data are reported in this protocol article.

## Discussion

This study investigates the effectiveness of a 16-week Plyo-HIIT program in improving MFR among children aged 9–12 years, compared to an SF program and an HE control. By focusing on MFR rather than weight or fat mass alone, the study targets a more meaningful health indicator for the prevention of childhood obesity. Traditional weight reduction strategies, such as caloric restriction or general aerobic programs, often lead to concurrent loss of fat and lean mass. This may compromise metabolic rate and physical function, increasing the risk of weight regain. Since skeletal muscle is critical for insulin sensitivity and resting energy expenditure, preserving or enhancing muscle mass while reducing fat may potentially offer more sustainable health benefits.

The Plyo-HIIT intervention is specifically designed to enhance neuromuscular adaptation, cardiovascular fitness, and metabolic function. Plyometric training improves muscular strength, bone health, and neuromotor development in children [21, 54, 55], while HIIT has been shown to improve body composition, aerobic capacity, and insulin sensitivity in children, including those with obesity [56, 57]. Although both modalities have been studied independently, their combined effects on regional muscle and fat distribution in children remain underexplored. This study aims to address this gap by evaluating the combined Plyo-HIIT protocol in a school-based environment.

To assess regional changes in body composition, MRI will be used to provide detailed measurements of muscle and adipose tissue distribution; this is more accurate than conventional methods [58, 59]. Secondary outcomes include assessments of muscular strength, endurance, cardiorespiratory fitness, physical activity levels, BMI, and cortisol stress markers, which are intended to provide a comprehensive summary of the physiological and psychosocial impact of the program.

Hair cortisol concentration, as a non-invasive biomarker, will be used to assess chronic physiological stress, facilitating investigation of the relationship between stress and obesity-related health outcomes in children. Elevated cortisol levels in children are linked to central adiposity, HPA axis dysregulation, insulin resistance, and emotional dysregulation [60, 61]. By integrating this biomarker with other health indicators, the study aims to generate a multidimensional assessment of the impact of the Plyo-HIIT intervention.

A three-arm randomized controlled design was used to allow comparison between the Plyo-HIIT program, the SF program, and the HE control. This design enables clear interpretation of intervention-specific effects. The primary outcome, MFR assessed by MRI, provides an objective and precise measure of body composition. Implementing the intervention within a school-based setting enhances its feasibility and may support broader application in public health. This pragmatic design aligns with routine practice and improves the generalizability of the findings to real-world settings.

The Plyo-HIIT program is designed to overcome common barriers to implementing physical activity in schools. These 45-minute sessions use body weight exercises, requiring no additional equipment, making them feasible within existing school facilities. This minimal equipment requirement of the program aligns with WHO recommendations for school-based moderate-to-vigorous physical activity, offering potential for widespread application both in Hong Kong, China and in other school settings. If successful, this study could support the implementation of Plyo-HIIT as a school-based program, contributing to local educational policies and health strategies aimed at promoting healthy body composition in children.

There are several limitations that should be acknowledged. Blinding of participants and instructors is not feasible, which may introduce performance bias. To mitigate this, outcome assessors will remain blinded, and standardized procedures will be strictly followed. Adherence to the program may be influenced by school schedules or student motivation. Therefore, attendance will be closely monitored, and motivational strategies such as certificates and engaging class formats will be employed to enhance participation. In addition, although MRI provides detailed composition data, its cost and accessibility may limit future large-scale use in routine school screening. Furthermore, although baseline MFR, age, and sex will be accounted for in the primary analysis, developmental variation across children aged 9–12 years, including differences related to biological maturation, may still influence body composition outcomes and should be considered when interpreting the findings.

Future studies may consider long-term follow-up assessments to evaluate whether the training effects are sustained over time. In addition, a cost-effectiveness analysis could be included to inform decisions about wider implementation in school health policies.

In summary, this RCT aims to demonstrate the effectiveness of the Plyo-HIIT program in improving MFR and overall physical health of school-age children. By focusing on both fat reduction and muscle preservation, the intervention addresses crucial metabolic and functional aspects of childhood health. The program’s design is well suited to real-world school environments and aligns with current physical activity guidelines, potentially establishing an evidence-based approach for childhood obesity prevention. This emphasis on improving body composition, rather than weight alone, may offer not only health benefits but also long-term protection against metabolic dysfunction.

## Data Availability

Data sharing is not applicable to this article as this is a protocol article and no datasets were generated or analyzed for the current manuscript.
